# Remodeling after acute myocardial infarction: mapping ventricular dilatation using three dimensional CMR image registration

**DOI:** 10.1186/1532-429X-14-41

**Published:** 2012-06-21

**Authors:** Declan P O’Regan, Wenzhe Shi, Ben Ariff, A John Baksi, Giuliana Durighel, Daniel Rueckert, Stuart A Cook

**Affiliations:** 1 Robert Steiner MRI Unit, MRC Clinical Sciences Centre, Imperial College London, Hammersmith Hospital Campus, Du Cane Road, London, W12 0NN, UK; 2 Department of Computing, Imperial College London, South Kensington Campus, Exhibition Road, London, SW7 2AZ, UK; 3 Department of Imaging, Imperial College Healthcare NHS Trust, Hammersmith Hospital, Du Cane Road, London, W12 0HS, UK; 4 Department of Cardiology, Imperial College Healthcare NHS Trust, London, Hammersmith Hospital, Du Cane Road, London, W12 0HS, UK

**Keywords:** Cardiovascular magnetic resonance, Acute myocardial infarction, Image analysis

## Abstract

**Background:**

Progressive heart failure due to remodeling is a major cause of morbidity and mortality following myocardial infarction. Conventional clinical imaging measures global volume changes, and currently there is no means of assessing regional myocardial dilatation in relation to ischemic burden. Here we use 3D co-registration of Cardiovascular Magnetic Resonance (CMR) images to assess the long-term effects of ischemia-reperfusion injury on left ventricular structure after acute ST-elevation myocardial infarction (STEMI).

**Methods:**

Forty six patients (age range 33–77 years) underwent CMR imaging within 7 days following primary percutaneous coronary intervention (PPCI) for acute STEMI with follow-up at one year. Functional cine imaging and Late Gadolinium Enhancement (LGE) were segmented and co-registered. Local left ventricular wall dilatation was assessed by using intensity-based similarities to track the structural changes in the heart between baseline and follow-up. Results are expressed as means, standard errors and 95% confidence interval (CI) of the difference.

**Results:**

Local left ventricular remodeling within infarcted myocardium was greater than in non-infarcted myocardium (1.6% ± 1.0 vs 0.3% ± 0.9, 95% CI: -2.4% – -0.2%, P = 0.02). One-way ANOVA revealed that transmural infarct thickness had a significant effect on the degree of local remodeling at one year (P < 0.0001) with greatest wall dilatation observed when infarct transmurality exceeded 50%. Infarct remodeling was more severe when microvascular obstruction (MVO) was present (3.8% ± 1.3 vs −1.6% ± 1.4, 95% CI: -9.1% – -1.5%, P = 0.007) and when end-diastolic volume had increased by >20% (4.8% ± 1.4 vs −0.15% ± 1.2, 95% CI: -8.9% – -0.9%, P = 0.017).

**Conclusions:**

The severity of ischemic injury has a significant effect on local ventricular wall remodeling with only modest dilatation observed within non-ischemic myocardium. Limitation of chronic remodeling may therefore depend on therapies directed at modulating ischemia-reperfusion injury. CMR co-registration has potential for assessing dynamic changes in ventricular structure in relation to therapeutic interventions.

## Background

Since the advent of primary percutaneous coronary intervention (PPCI) to treat acute myocardial infarction immediate survival has improved but at the expense of a rising incidence of progressive heart failure [1]. Ischemia-reperfusion injury leads to a sequence of events that result in predictable changes to the structure and function of the left ventricle (LV) that may eventually cause congestive heart disease [2]. Our understanding of remodeling is largely based on animal models which show that within the first few days after coronary occlusion there is slippage and stretching of myocytes in the infarcted zone [3]. Late remodeling also involves changes to the non-ischemic myocardium as it adapts to the extra load placed on it by dilating [4,5]. Current methods of evaluating remodeling rely on measuring global changes in left ventricular volume, however this does not reveal where myocardial enlargement and dilatation occur within the ventricle or how remodeling is influenced by the severity of local ischemic injury. Determining the pattern of left ventricular remodeling in patients and the contribution made by infarcted and remote myocardium to chamber dilatation has importance for evaluating interventions aimed at preventing heart failure [6].

In this paper we apply techniques previously developed in neuroscience research to align and co-register Cardiovascular Magnetic Resonance (CMR) images of the heart to map the effects of remodeling in three dimensions [7]. This framework enables highly consistent comparisons to be made between different CMR sequences obtained at various time-points [8]. By tracking how each point on the myocardial surface changes relative to neighboring points we can build a three dimensional model of how the LV remodels over time. Co-registering structural imaging with late-enhancement sequences also allows the extent of local remodeling to be compared to the degree of ischemic injury present. However, developing these techniques in the heart is challenging as it requires a consistent and accurate approach to co-registering and transforming the cardiac MR images acquired in a longitudinal study.

The aim of this study was to use 3D co-registration techniques to determine how the LV remodels after reperfused acute ST-elevation myocardial infarction (STEMI) and the relationship to ischemic injury.

## Methods

### Participants

The study was approved by the Hospital’s research ethics committee and all patients gave written informed consent. To be included in the study each patient had to have been admitted within 24 hours of the onset of chest pain with an ECG diagnosis of acute STEMI and angiographically proven partial or complete coronary occlusion of the infarct-related artery. Exclusion criteria were contraindications to CMR, previous MI or heart failure, clinical instability, significant arrhythmias, pregnancy or lactation. In total MR images from 46 patients were analyzed (44 male, 2 female; age range 33 to 77 years; mean age 55 years). A baseline CMR was performed within 1 week of PPCI and the follow-up study at one year.

### Coronary intervention

Coronary catheterization was used to identify and treat the infarct-related artery and all patients received either a bare-metal or drug-eluting stent. Standard medical treatment was provided. Flow in the infarct-related artery was graded using the Thrombolysis in Myocardial Infarction trial (TIMI) criteria prior to and after intervention [9].

### CMR protocol

The CMR studies were performed on a 1.5 T Philips Achieva system (Best, Netherlands) using a 32-channel coil. Myocardial edema was imaged with a navigator-gated black blood T2-weighted turbo spin echo sequence with spectrally selective inversion recovery (SPIR) fat suppression using the following parameters - field of view 350 x 280 mm; matrix, 256 x 123; repetition time msec/echo time msec, 2 R-R intervals/100; in-plane resolution, 1.4 x 2.3 mm; section thickness, 8 mm. An intravenous bolus of Gadobutrol 1.0 mmol/ml (Gadovist; Bayer Schering Pharma, Berlin, Germany) was administered at a dose of 0.15 mmol/kg. Early enhancement imaging was performed to assess microvascular obstruction (MVO) with a single breath hold 3D inversion recovery sequence within 1 minute of contrast injection using the following parameters - field of view, 350 x 290 mm; matrix 252 x 135; repetition time msec/echo time msec, 4.4/1.3; in-plane resolution, 1.4 x 2.1 mm; section thickness 8 mm. Late Gadolinium Enhancement (LGE) was performed to assess infarct size 10 minutes after contrast injection with a 2D inversion recovery sequence using the following parameters - field of view, 320 x 320 mm; matrix 200 x 147; repetition time msec/echo time msec, 5.5/2.5; in-plane resolution 1.6 x 2.2 mm; section thickness 8 mm. Balanced-steady state free precession (b-SSFP) images in the left ventricular short axis plane were acquired using the following parameters - field of view 320 x 320 mm; matrix, 160 x 151; repetition time msec/echo time msec, 3.0/1.5; shot duration, 50 ms; in-plane resolution, 2.0 x 2.2 mm; number of cardiac phases, 30; section thickness, 8 mm.

### Image segmentation and registration

**Figure 1:**
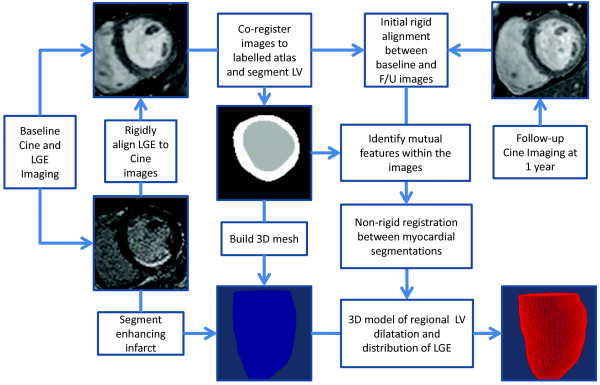
**Flow chart showing the steps in image co-registration.** The cardiac atlas labels are co-registered to the baseline cine and LGE sequences to segment the myocardium. The infarct itself is then segmented using a Gaussian mixture model. The baseline and follow-up cine images are first rigidly aligned for anatomical consistency. A non-rigid registration is then used to build a map of local ventricular dilatation by tracking the relative position of intrinsic landmarks shared by the images.

**Figure 2:**
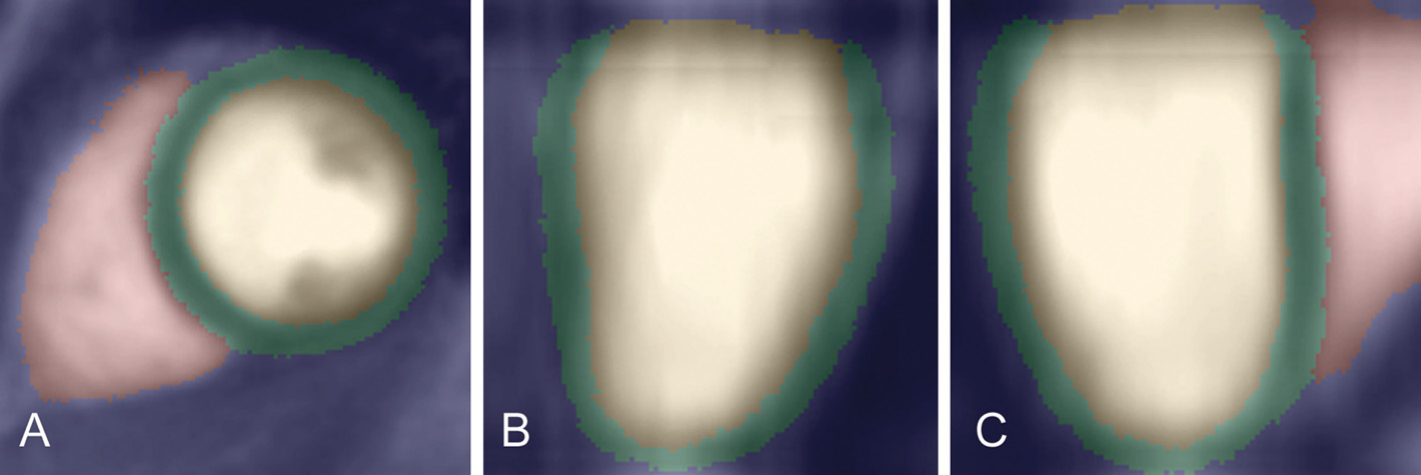
**Probabilistic cardiac atlas.** A labeled probabilistic atlas was used to provide prior knowledge of the cardiac anatomy during the segmentation process. Multiplanar reconstructions of the atlas are shown where each voxel is assigned a probability of belonging to left ventricular blood pool (yellow), left ventricular wall (green), right ventricle blood pool (red) and background (blue). (**A**) Left ventricular short axis. (**B**) Vertical long axis. (**C**) Horizontal long axis.

Our approach was to co-register the cine and LGE images onto an atlas template, and then use intensity-based similarities within the heart to track local expansion of the LV in the first year after acute STEMI (Figure 1). The atlas comprised a set of manually segmented end-diastolic images where each voxel is assigned a probability of being blood pool, left ventricular wall, right ventricle blood pool or background structures (Figure 2) [10]. The prior information in the atlas was used in the segmentation of the target images and to correct for differences in alignment between individual subjects and MR acquisitions. The atlas was registered to the target image using affine, local affine and then non-rigid registration [11]. We used an expectation-maximum (EM) technique with spatial information from the probabilistic atlas to segment the LV from surrounding structures [10]. The accuracy of the automatic segmentation was assessed by manually defining the endocardium and epicardium on the same image sets and quantifying the mean surface-to-surface distances between the respective segmentations. The baseline LGE images were then rigidly aligned to the baseline cine short axis stack to co-register structural imaging with the distribution of infarcted tissue. We used a two-component Gaussian mixture model (GMM) to discriminate between enhancing and non-enhancing voxels on the LGE images [12]. The distribution with a higher mean signal intensity mean was considered as the infarct intensity distribution. The transmural extent of infarction was calculated as the proportion of infarcted myocardium in the radial direction of the left ventricular wall. Wall thickness was calculated along lines joining an epicardial vertex to the nearest endocardial vertex.

**Figure 3:**
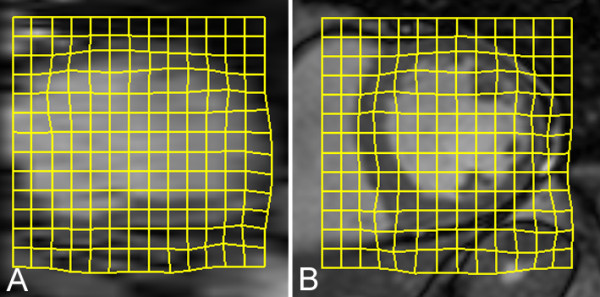
**Image transformation between time-points.** A non-rigid transformation (yellow grid) between the baseline cine images and those obtained at follow-up is used to assess regional remodeling in three dimensions. Intrinsic features within the image are used to track how each part of the ventricular wall deforms and dilates over time as a consequence of infarction. (**A**) Long axis. (**B**) Short axis.

**Figure 4:**
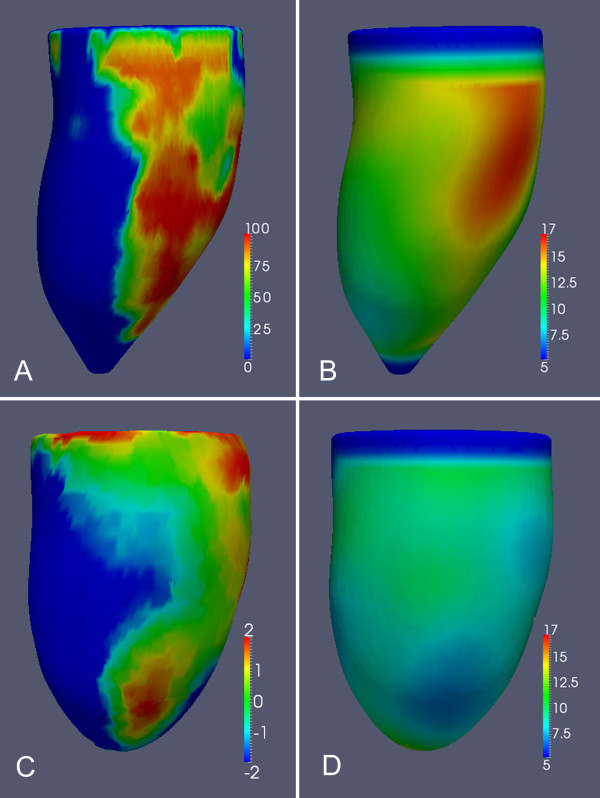
**(A - D). 3D models of the left ventricle in an 51 year old male patient following primary percutaneous coronary intervention (PPCI) to a left circumflex artery occlusion.** The images show co-registered data from segmented cine and Late Gadolinium Enhancement (LGE) images. (**A**) Distribution of enhancing necrosis (transmural percentage of infarcted myocardium) at baseline. (**B**) Left ventricular wall thickness (mm) at baseline. (**C**) Local myocardial remodeling between baseline and follow-up (percentage expansion). (**D**) Left ventricular wall thickness at follow-up (mm). These images demonstrate the anatomic relationship between the zone of infarction and the extent of local remodeling and wall thinning.

A 3D mesh reconstruction of the endocardial and epicardial surfaces was created from the baseline segmentation [13,14]. To make longitudinal comparisons the baseline and follow-up reconstructions were first rigidly aligned using intensity information from the whole image to achieve anatomical consistency. A non-rigid registration between the segmentations at each visit was then used to measure how the ventricle deforms over time by tracking the relative position of corresponding points in the myocardium. This approach uses normalized mutual information in the images to identify matching anatomical features and signal intensity patterns to use as landmarks (Figure 3) [11]. As the LV remodels and the myocardium expands the relative separation of corresponding points on the mesh reconstruction increases (Figure 4). Local ventricular remodeling was defined as the mean change in separation between each vertex on the mesh and its surrounding neighbors [5,15] and was therefore independent of infarct size.

The presence of MVO was defined as a region of low signal intensity within the infarcted region on early enhancement images [16]. Left ventricular mass, end diastolic volume and end systolic volume were measured using CMRtools (Cardiovascular Imaging Solutions, London, UK). Global left ventricular remodeling was considered to be present if there was at least a 20% increase in end diastolic volume between baseline and follow-up [17].

### Statistical analysis

Data were analyzed with IBM SPSS statistics (IBM, Chicago, Ill). Remodeling data are presented as mean and standard error of the mean. Comparisons were made using two tailed t-tests and reported as a P value with the 95% confidence interval (CI) of the difference of the mean. The effect of infarct thickness on remodeling was evaluated with a one-way analysis of variance (ANOVA). A P value < 0.05 was considered significant. As this was a preliminary study no correction for multiple tests was made.

## Results

Baseline characteristics for all patients are shown in Table 1. The baseline CMR was performed at a mean of 2.7 days (range 1 – 7 days) post-PPCI and the follow-up study at a mean of 54 weeks (range 53 – 57 weeks). All subjects demonstrated an enhancing infarct on the LGE images within the vascular territory of the culprit coronary artery corresponding to high signal on T2-weighted imaging. No patients demonstrated co-existing chronic infarctions or infarcts in multiple coronary territories. Mean infarct size was 13.3% (range 1.2 - 34.0%). Changes in left ventricular functional indices are shown in Table 2. Twenty seven (59%) patients had evidence of MVO on the baseline LGE images, and in 16 (35%) patients there was global LV remodeling. At follow-up 39 patients were NYHA class I, 4 patients class II, 2 patients class III and 1 patient class IV.

**Table 1:** Baseline characteristics of the patients. Stratified by which patients developed remodeling at follow-up

	Remodeled	Non-remodeled	All
Age (y)	54±12	53±10	55±10
Sex			
Male	16	28	44
Female	0	2	2
Body Surface Area (kg/m^2^)	1.91±0.33	1.96±0.2	1.97±0.18
Systolic blood pressure (mmHg)	131±32	128±28	133±28
Diastolic blood pressure (mmHg)	83±22	85±22	84±20
Heart Rate (min^-1^)	73±18	73±13	75±16
Diabetes mellitus (%)	19%	13%	15%
Current or ex-smoker (%)	44%	67%	59%
Hypertension (%)	38%	37%	37%
Time from symptoms to reperfusion (hours)	3.5±2.6	3.4±3.0	3.5±2.6
Peak Creatinine kinase (IU/L)	2216±1994	1775±1581	2240±2034
Peak Troponin I (μg/L)	65.0±78.7	63.6±92.2	64±80
Culprit coronary artery (%)			
Left anterior descending	56	53	54
Circumflex artery	6	17	13
Right coronary	38	30	33
TIMI flow Pre-PPCI (%)			
Grade 0	94	80	85
Grade I	6	3	9
Grade II	0	7	4
Grade III	0	0	0
TIMI flow Post-PPCI (%)			
Grade II	6	0	4
Grade III	63	100	96
Discharge medication (%)			
Aspirin	100	100	100
Clopidogrel	96	96	96
Beta blocker	94	90	92
Ca^2+^ channel blocker	6	0	2
Angiotensin converting enzyme inhibitor	98	94	96
Statin	98	98	98

Values are means ± standard deviations. *PPCI* = Primary percutaneous coronary intervention. *LV* = Left Ventricle.

**Table 2:** Comparison of left ventricular functional indices obtained at baseline and 1 year follow-up

	Baseline	Follow-up	P value
LV mass (g)	155 ± 44	133 ± 33	<0.001
LV end diastolic volume (ml)	146 ± 38	160 ± 46	0.004
LV end systolic volume (ml)	67 ± 26	71 ± 31	0.4
Stroke Volume (ml)	80 ± 19	90 ± 28	0.006
Ejection Fraction (%)	56 ± 9	57 ± 12	0.18

Values are mean ± standard deviations. *LV* = Left Ventricle.

The mean surface-to-surface distance between manual and automatic segmentations was 1.2 mm ± 0.3 for the endocardium and 1.5 mm ± 0.2 for the epicardium.

### Local left ventricular remodeling

**Figure 5:**
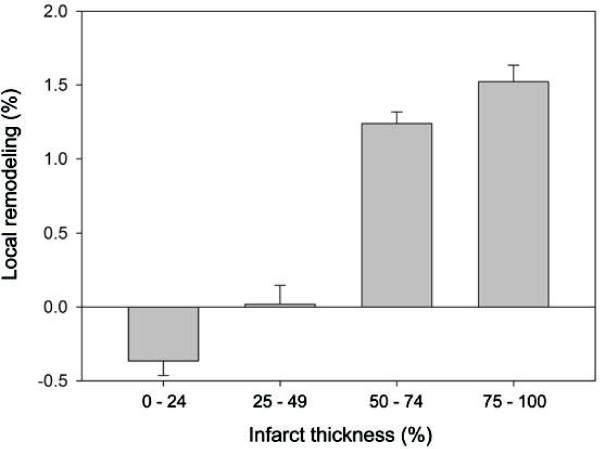
**The relationship between local remodeling and infarct transmurality (Mean values with 95% confidence interval).** The transmural extent of infarction at baseline has a significant influence on the severity myocardial remodeling at the same location in the left ventricle (ANOVA P < 0.0001). Local ventricular expansion is most marked when ≥50% of the wall is infarcted.

Remodeling within infarcted myocardium was greater than in remote myocardium (1.6% ± 1.0 vs 0.3% ± 0.9, CI: -2.4% – -0.2%, P = 0.02). One-way ANOVA revealed that infarct thickness had a significant effect on the degree of local remodeling at one year (P < 0.0001) (Figure 5) with wall dilatation observed when infarct transmurality exceeded 50%. Local remodeling was more severe when infarcts contained regions of MVO (3.8% ± 1.3 vs −1.6% ± 1.4, CI: -9.1% – -1.5%, P = 0.007) and when end-diastolic volume had increased by >20% (4.8% ± 1.4 vs −0.15% ± 1.2, CI: -8.9% – -0.9%, P = 0.017).

### Wall thickness

Mean wall thickness averaged throughout the acutely infarcted region decreased significantly during the first year (10.1 mm ± 0.3 vs 8.9 mm ± 1.7, CI: 0.5 – 2.0, P = 0.002) and was unchanged in the remote region (7.1 mm ± 0.2 vs 7.1 mm ± 0.2, CI: -0.5 – 0.5, P = 0.95). There was no difference in infarct wall thickness when either global left ventricular remodeling (9.3 mm ± 0.5 vs −8.7 mm ± 0.3, CI: -1.7 – 0.4, P = 0.24), or MVO was present (9.0 mm ± 0.3 vs −8.8 mm ± 0.4, CI: -1.2 – 0.9, P = 0.75).

## Discussion

In this study we have used image-registration to map in three dimensions where the left ventricular wall dilates in the first year following STEMI and to relate this to the extent of local ischemic injury. Our findings show that following PPCI with standard medical therapy infarct transmurality and MVO have a significant effect on local ventricular wall remodeling while only modest dilatation is observed within non-ischemic myocardium.

Image co-registration is an emerging technique for the statistical analysis and quantification of cardiac motion and geometry [8]. In this study we used a labeled atlas to provide prior knowledge of cardiac anatomy to assist with image segmentation and as a template onto which different MR sequences are co-registered. This approach has been successfully used for determining shape variation of the left ventricle within patient populations [18,19]. It is also applicable to analyzing dynamic changes in cardiac morphology by using intrinsic signal intensity variations and anatomical features in the heart to track myocardial deformation over time - a process analogous to the explicit tags used in strain imaging [20,21]. In this work we used normalized mutual information between the myocardial segmentations at baseline and follow-up to map the chronic regional structural adaptations that occur within the left ventricle in response to STEMI. We also co-registered the anatomical images with the infarct segmentation enabling us to compare the severity of local remodeling with the extent of transmural necrosis at each point in the LV. This approach provides an appealing new methodology for assessing temporal changes in left ventricular morphology in relation to local ischemic injury.

A limitation of conventional imaging is that assessment of cavity volumes does not reflect the dynamic regional changes in myocardial structure that occur in response to ischemia-reperfusion injury. In animal studies of remodeling there is an acute phase of lateral slippage of the myocardial planes within the infarct leading to progressive left ventricular dilatation [22]. In the later stages of disease non-ischemic myocardium may develop a maladaptive response of ventricular dilatation which ultimately leads to heart failure [5,23]. Left ventricular remodeling is also determined by factors other than initial infarct size [17,24] and both infarct transmurality and the presence of persistent MVO influence the development of chronic heart failure [25-27]. Despite revascularization and medical therapy 35% of the patients in our study developed global remodeling as defined by end diastolic volumes. Our findings indicate that infarcted myocardium plays a much greater role in such chronic remodeling than non-ischemic tissue. Our observation of progressive dilatation in the ischemic territory is consistent with elongation of the infarcted myocardium which typically develops during early post-infarction remodeling [28]. The extent of transmural necrosis is known to influence global remodeling [27] and we observed that local myocardial dilatation occurs when infarct thickness exceeds 50%. This is consistent with findings that acute sub-endocardial infarctions remain viable with the potential for contractile recovery [29]. We also observed the highest rates of local wall dilatation when infarcts had regions of MVO, which is recognized as a predictor of adverse remodeling [16]. We observed a significant decrease in left ventricular mass and wall thickness in the infarcted region over time and this is consistent with transient ischemia-reperfusion edema within the infarct zone [30].

In our cohort remodeling in the non-ischemic territory was modest compared to the infarcted region. The late phase of ventricular remodeling has been shown to be amendable to medical therapy, as chronic administration of angiotensin-converting enzyme inhibitors (ACEIs) are associated with a reduction in the extent of ventricular dilation [4,31] and prolonged therapy with β-blockers may limit or even reverse remodeling and dysfunction [32,33]. The great majority of patients in this study were prescribed both ACEIs and β-blockers and our findings suggest that this therapy may be effective in limiting the development of maladaptive remodeling within remote myocardium following successful PPCI.

Image registration offers a more refined model of left ventricular adaptations to infarction than can be derived from global measures of cavity volume. The potential clinical applications include interventional studies of cardio-protective therapies where the physiological effects of remodeling within infarcted and remote myocardium may be separately measured and compared to imaging markers of ischemic injury. The approach may also be helpful for predicting the complications of ischemic remodeling such as ventricular aneurysm formation and the development of ischemic mitral regurgitation. Developments in parallel imaging also make 3D acquisition of both cine images and LGE appealing for registration techniques as they avoid section mis-registration [34-36].

We do not have inter-study reproducibility data for the registration technique as the patients were only scanned once at each visit. We used the segmentation of the myocardium to guide registration so the final transformation is weighted to geometric changes within the myocardium. We also used an initial non-rigid transformation to limit the influence of local structures other than myocardium. However, as the mutual information between images is not explicitly defined the registration may also be influenced by structures immediately adjacent to the heart. EM segmentation provided an accurate segmentation of the myocardial boundaries, but the influence of signal intensity differences in acute and remote myocardium was not evaluated. Mean wall thickness in infarcted myocardium at follow-up was 8.9 mm and so there were typically at least 4 voxels across the width of the LV to perform the non-rigid registration However, accuracy of registration may be limited by severely thinned myocardium. We did not use follow-up LGE images in the remodeling analysis as we wanted to distinguish expansion of remodeled myocardium from expansion of the zone of necrosis. We also did not segment the T2 images due to the relatively low signal to noise ratio of these sequences.

In our cohort the mean baseline LVEF was not significantly impaired and this is likely to have been influenced by the exclusion of subjects with acutely impaired left ventricular function who were not suitable for CMR within 7 days of PPCI. However, the observation that local remodeling may be observed despite a normal ejection fraction supports the premise that global indices of function may mask the regional effects of ischemic damage. Further research is needed to explore whether local morphological changes affect long-term outcome or are independent predictors of heart failure. GMM has not been formally assessed for infarct segmentation although the methodology is similar to validated techniques for pixel-weighted infarct quantification [37]. Our findings are limited to assessing structural remodeling and we did not measure regional strain or wall thickening to evaluate changes in contractile function as a consequence of STEMI [38]. Further work is also needed to establish the correspondence between image-based transformations and ultra-structural adaptations within the myocardium.

## Conclusions

The severity of ischemic injury has a significant effect on local ventricular wall remodeling while only modest dilatation is observed within non-ischemic myocardium. This indicates that revascularization and standard medical therapy may be effective at preventing structural changes in the remote myocardium, and that further limitation of chronic remodeling may depend on therapies directed at modulating ischemia-reperfusion injury.

Co-registration of CMR images acquired in longitudinal studies is an analysis technique which has potential for assessing dynamic changes in ventricular structure in relation to therapeutic interventions.

## Abbreviations

AAR, Area-at-risk; ACEI, Angiotensin-converting enzyme inhibitor; CMR, Cardiac magnetic resonance; LGE, Late gadolinium enhancement; MVO, Microvascular obstruction; NYHA, New York Heart Association Functional Classification; PPCI, Primary percutaneous coronary intervention; STEMI, ST-segment elevation myocardial infarction; T2-SPIR, T2 weighted spectrally selective inversion recovery; TIMI, Thrombolysis in Myocardial Infarction trial grading.

## Competing interests

The authors declare that they have no competing interests.

## Authors’ contributions

DPO’R conceived of the study design, performed the statistical analysis and drafted the manuscript. WS developed the co-registration methods and analyzed the data. BA participated in the coordination of the study and revising the manuscript. AJB contributed to the manual data analysis and revising the manuscript. GD scanned the patients, contributed to data analysis and revised the manuscript. DR participated in the study design and in developing the image analysis methods. SAC conceived of the research program, coordinated the study and revised the manuscript. All authors read and approved the final manuscript.
